# Effect of *Cucurbita Maxima* on Control of Blood Glucose in Diabetic Critically Ill Patients

**DOI:** 10.15171/apb.2018.040

**Published:** 2018-06-19

**Authors:** Ata Mahmoodpoor, Mahsa Medghalchi, Hossein Nazemiyeh, Parina Asgharian, Kamran Shadvar, Hadi Hamishehkar

**Affiliations:** ^1^Department of Anesthesiology, Tabriz University of Medical Sciences, Tabriz, Iran.; ^2^Iranian Evidence Based Medicine Center of Excellence, Tabriz University of Medical Sciences, Tabriz, Iran.; ^3^Research Center for Pharmaceutical Nanotechnology, Tabriz University of Medical Sciences, Tabriz, Iran.; ^4^Department of Pharmacognosy, Faculty of Pharmacy, Tabriz University of Medical Sciences, Tabriz, Iran.; ^5^Student Research Committee, Tabriz University of Medical Sciences, Tabriz, Iran.; ^6^Cardiovascular Research Center, Tabriz University of Medical Sciences, Tabriz, Iran.; ^7^Drug Applied Research Center, Tabriz University of Medical Sciences, Tabriz, Iran.

**Keywords:** *Cucurbita maxima*, Blood glucose, Hyperglycemia, Critical illness, Intensive care unit

## Abstract

***Purpose:*** Cucurbita maxima Duchense (C. maxima) has been widely used in China and Mexico as a hypoglycemic plant for controlling blood glucose in diabetic patients. Furthermore, in northwest of Iran, this plant is used traditionally for controlling of diabetes. We examined the effect of C. maxima pulp besides insulin on control of hyperglycemia in diabetic patients admitted to Intensive care unit (ICU).

***Methods:*** Twenty critically ill patients who were admitted to the ICU were enrolled in this study. 5g lyophilized powder of C. maxima was administrated every 12 hours for 3 days. Moreover, blood glucose level and insulin dose were measured every 1-4 hours during 3 days before administration and 3days at the time of C. maxima administration.

***Results:*** The average of glucose level in 3 days before C. maxima administration was 214.9 ± 55.7 mg/dl, while in 3 days during C. maxima administration it was decreased to 178.4 ± 36.1 mg/dl (P<0.001). Additionally, the average insulin dose during 3 days before intervention was 48.05 ± 36.5 IU and during the 3 days of C. maxima administration was decreased to 39.5 ± 27.8 IU (P=0.06).

***Conclusion:*** It seems that C. maxima may decrease high blood glucose level fast and effective in diabetic critically ill patients.

## Introduction

Hyperglycemia frequently occurs after injury or critical illness in diabetic patients. Prevalence of Insulin ‎resistance in the most of critically ill patients causes impairment in the blood glucose control.^1^ ‎Stress mediators like stress hormones, cytokines and the central nervous system are involved in ‎carbohydrate metabolism particularly in the liver and skeletal muscle.^2^ ‎Systemic catecholamine release, cytokine release following systemic inflammation and direct ‎systemic stimulation can all result in hepatic glycogenolysis and finally hyperglycemia.^3^ In septic patients, pathological activation of the innate immune system can induce insulin resistance ‎and hyperglycemia.^4^ As a result, development of ‎hyperglycemia and insulin resistance is more common in patients admitted to Intensive Care Unit (ICU). Hyperglycemia ‏has been identified as an independent risk factor for undesirable outcomes in critically ill patients and it can ‎cause complications including severe infections, polyneuropathy, multiple organ failure and furthermore ‎death.^5,6^

In intensive care setting, using of oral anti-diabetic medications from Biguanide and Thiazolidinedione family is ‎limited. In addition, Metformin is contraindicated in patients with sepsis, metabolic acidosis, renal failure,‏ and unstable hemodynamic because lactic acidosis may be developed as a fatal consequent.^7^ Peripheral edema is ‎common with Pioglitazone particularly in patients suffering from congestive heart failure.^8^ These complications are common among patients admitted to ICU. Hence, introducing a drug augmenting insulin sensitivity and better control of blood glucose in such patients seems to be valuable. In this regard, using herbal products is increasing.^9,10^ *Cucurbita maxima* Duchense (*C. maxima*) (English name: Winter squash) has been known as a medical plant; it ‎belongs to the family of Cucurbitaceae.^11,12^ *C. maxima* is a dicotyledonous seed vegetable. It has flexible succulent stem with trifoliate, alternate and also leafstalk leaves.^11,13,14^ The biological active components of *C. maxima* fruit include polysaccharides, para aminobenzoic acid, fixed oils, proteins and peptides^13,15^ sterol, flavonoids^16^ tannins, phenolics and saponins.^17^ In addition, pectin, as a water- soluble fiber is a common and main compound of pumpkin plants.^18^ Several pharmacological activities such as anti-oxidant, anti-diabetic properties,^19,20^ anti-hyperlipidemic, hepatoprotective^21^ and anti- cancer,^22^ have been reported by different species of these plants. The active hypoglycemic components of *C. maxima* have been found in the fruit pulp^13^ and seeds^11^ which defined to be ‎beneficial for the diabetic rats and also human who are type2 diabetic patients. Among 200 Chinese medical plants which have known as anti-diabetic plants, *C. maxima* and *C. moschata* have been frequently used.^23^ Moreover, its use for diabetes was reported from Mexico^16^ Since there is no information regarding the effect of *C. maxima* on blood glucose control levels in patients admitted to ICU, the objective of this preliminary study was to examine the effect of *C. maxima* pulp besides insulin on glucose level in diabetic patients admitted to ICU.

## Materials and Methods

### Preparation of winter squash powder

The fruits of *C. maxima* were collected from East Azarbaijan province (Iran) during November 2013. After matching with available references by professional herbalis, round Pepo fruit and developed tendril had been considered for *C.maxima* with voucher number as CV. Buttercup squash TBZ-fph 1713. and also, retained in the herbarium of the Faculty of Pharmacy, Tabriz University of Medical Sciences, Iran. The fruits were thoroughly washed with potable water and cleanser then, were sliced in little pieces to be ‎processed in an electric domestic extractor. After obtaining the juice, the volume reduced in ‎rotary evaporator in 37°C. After that, the juice was powdered using freeze dryer, and stored at 4°C for 4 ‎months.‎

### Participants

This was an uncontrolled before-after clinical trial has been performed at two ICUs of two hospitals affiliated to Tabriz University of Medical Sciences. ‏This study included the patients were admitted to ICUs between the first of January 2014 to May 2016. Patients aged 18 years or older and also, were diabetic with lack of appropriate response to insulin therapy (their blood glucose levels were higher than 160 mg/dl in spite of insulin therapy used on sliding scale in therapy for 24h), have been entered into the study.^24^ 12 patients had diabetes type 1 and 8 patients had type 2 diabetes received insulin in their Medicinal diet. We excluded patients with no evidence of diabetes ‎from diabetic patients with controlled blood glucose levels using insulin therapy. The study protocol ‎was approved by the ethic committee of the Tabriz University of medical science and was recorded in the International Clinical Trials Registry Platform (ICTRP) with identifier IRCT201311118307N2.

### Study Protocol

In the study of Acosta-Patin˜o and colleagues^25^ who performed on type 2 diabetes, the dose of *Cucurbita ficifolia* was 4 ml/kg of fruit juice. Accordingly, and considering the average weight of 70 kg for patients, after drying 280 ml of juice in oven, the amount of dry matter remained was 9 grams. Therefore, in this study, the dose of *Cucurbita maxima* was assumed to be 10 grams per day in two divided doses. This dose is close to that of the plant used in traditional medicine (one glass of *C. maxima* fruit juice every day)

The patients received 5 g of the *C. maxima* powder every 12 hours for 3 days. Blood levels of glucose and the dose of insulin were measured every 1-4 hours depending on severity of hyperglycemia. The data were collected during 3 days before and within 3 days of *C. maxima* administration.

For all patients, Acute Physiology and Chronic Health Evaluation (APACHE II) and Sequential Organ Failure Assessment score (SOFA) were calculated daily to determine the severity of primary disease and organ failure, respectively.

### Statistical Analysis

We are planning a study of a continuous response variable from matched pairs of study subjects. Prior data from a pilot study with 6 patients have indicated that the difference in the response of matched pairs is normally distributed with standard deviation of 29.5. The difference in the mean response of matched pairs was 30, and then the sample size calculated to be 12 pairs of subjects to be able to reject the null hypothesis that this response difference is zero with probability (power =0.9). The Type I error probability associated with the test of null hypothesis is 0.05. For increasing the power of study, we included 20 patients in our study. Paired-samples t-test was used to compare mean blood glucose level 3 days before administration of *C. maxima* with 3 days of *C. maxima* administration and repeated measures analysis of variance was used to detect significant changes in the blood glucose and prescribed insulin for patients during sequential measured times. Greenhouse-Geisser test was used when Mauchly's test of sphericity was significant to test the significant changes of blood glucose and insulin within group. A two-sided P value less than 0.05 was considered significant. All statistical analyses were performed with the SPSS software (Statistical Package for the Social Sciences, version 16.0, SPSS Inc, Chicago, Ill, USA).

## Results

A total of 20 patients, 11 males and 9 females, aged 45-82 years, enrolled in the study with a follow-up period of 6 days. Table 1 shows the characteristics and disease severity for enrolled patients. The medications that patients received during the study and affecting blood glucose were listed in the Table 1. Eight patients received beta blocker and fluoroquinolones, 5 patients beta blocker, 4 patients fluoroquinolones, 2 patients corticosteroids and one patient received beta blocker and corticosteroids

**Table 1:** Severity of disease and characteristics of patients*

Age, years (min-max)	65.4 ± 10.9 (45-79)
Sex, Male no (%)	11 (55%)
SOFA (admission)	5.1 ± 2.1
SOFA (mean of 3 days before *C. maxima* Administration)	4.9 ± 1.6
SOFA (mean of 3 days of *C. maxima* Administration)	5.08 ± 2.24
APACHE II (admission)	18.8 ± 6.9
APACHE II (mean of 3 days before *C. maxima* Administration)	18.4 ± 6.5
APACHE II (mean of 3 days of *C. maxima* Administration)	18.5 ± 7.8
GCS (admission)	10.2 ± 3.3
HA1c	9.35 ± 2.5
Drugs, number of patients	
Beta Blocker	14
Corticosteroids	3
Fluoroquinolones	12

*Values are shown as the percentage (number) or mean ± standard deviation; SOFA: Sequential Organ Failure Assessment score; APACHE II: Acute Physiology and Chronic Health Evaluation; *C. maxima*: *Cucurbita maxima*; GCS: Glasgow Coma Scale

There were no significant changes in the average of APACHE II and SOFA scores following the administration of *C. maxima*. (P>0.05).

The effect of *C. maxima* on blood glucose levels and infused insulin dose is shown in Table 2.

**Table 2:** Blood glucose levels and insulin dose in diabetic patients admitted in ICU before and after the oral administration of *C. maxima* powder

**Day**	**BS (mg/dL)**	**Ins.Dose (unit)**
Before *C. maxima* administration		
-3	209.2 ± 41.3	36.7 ± 31.1
-2	218.2 ± 66.2	49.4 ±37.8
-1	217.4 ± 59.1	58.01 ± 38.8
Mean	214.9 ± 55.7	48.05 ± 36.5
After *C. maxima* administration		
1	182.6 ± 33.1	43.6 ± 31.8
2	175.1 ± 28.1	40.3 ± 26.9
3	177.4 ± 46.3	34.9 ± 25.1
Mean	178.4 ± 36.1	39.5 ± 27.8
MD (95% CI), Pv*	(22.5 – 50.6), <0.001	(-5.2 – 17.2), 0.28

BS: blood sugar, Ins: insulin, MD: Mean Difference, CI: Confidence Interval, *C. maxima*: *Cucurbita maxima* *Results based on Paired T test comparing mean blood glucose level before and after *C. maxima* administration

**Figure 1:**
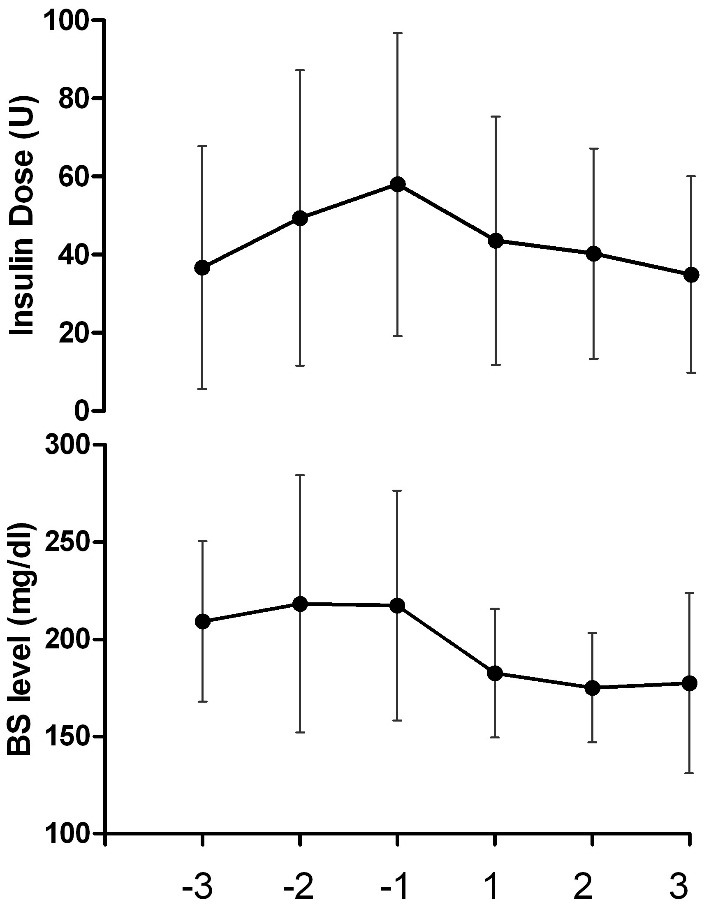
BS; Blood Sugar. Changes in consumed insulin dose and blood glucose levels during 6 days of study. -3 to -1 implies 3 days before intervention and 1 to 3 show 3 days during *C. maxima* powder prescription for patients.

The average glucose level in 3 days before *C. maxima* administration was 214.9 ± 55.7 mg/dl and in 3 days after that blood glucose level decreased to 178.4 ± 36.1 mg/dl. The amount of decrease was significant compared with the pre- *C. maxima* administration level (P<0.001). In addition, repeated measurements analysis for blood glucose levels during the study days showed significant decrease (P= 0.002) (Figure1). Pairwise Comparisons among average glucose level in 3 days before *C. maxima* administration and blood glucose level in each 3 days after *C. maxima* administration showed statistically significant differences (p<0.05).

The average dose of infused insulin during 6 days has been summarized in Table 2. In first 3 days, the average insulin dose was 48.05 ± 36.5 IU and during the last 3 days, it decreased to 39.5 ± 27.8 IU (P=0.06). There were not any significant differences among the average dose of infused insulin during 3 days before *C. maxima* administration and the average insulin doses in each 3 days after *C. maxima* administration (p>0.05).

## Discussion

Results of the present study indicated that administration of 5g *C. maxima* powder twice daily for 3days in diabetic patients admitted to ICU significantly reduced blood glucose level as compared to that in 3days before *C. maxima* administration. In addition, this plant was able to decrease insulin dose, though it was insignificant. Our results were in line with Asghary et al,^26^ they showed that, treating diabetic ‎rats by other species of *Cucurbita* fruit powder (*C. pepo*) reduced blood glucose ‎level significantly and increase the blood insulin level. The results of that study demonstrated blood glucose decreasing effect of Pumpkin was as well as ‎glibenclamide. ‎ Furthermore, other study,^11^ has demonstrated the effect of different extracts of *C. maxima* seeds on decreasing the level of blood glucose concentration in streptozotocin induced diabetic rats.

‏Previous studies have indicated that Protein-bound polysaccharides (PBPs) found in other species of *Cucurbita* have anti-diabetic properties. Li et al^27^ carried out examination to evaluate the effect of orally administered different ‎doses of PBPs of pumpkin on diabetic rats. In ‎mentioned study, PBPs indicated hypoglycemic activity, even stronger than that ‎observed with 20 mg/kg of glibenclamide. Based on the results of that study, it can be suggested that different doses of PBPs may have hypoglycemic effect. The phytochemical investigation indicated the different extracts of *C. maxima* contain carbohydrates, flavanoids, tannins, phenolics, pectines and saponins.^17^ Pumpkin polysaccharides could increase anti-oxidant capacity by elevating glutathione peroxidase and superoxide dismutase activity and also, decreasing the malonaldehyde in mice serum.^28^ On the other hand, flavonoides (like quercetine) and saponines^26-30^ have antioxidant activities which play a main role in anti-diabetic effect of pumpkin especially in critically ill patients who oxidative stress may cause insulin resistance and diabetes. Additionally, it has shown that^31^ pumpkin’s polysaccharides and pectines increase the serum insulin levels and reduce blood glucose.

In a clinical study,^25^ ten Type 2 diabetic patients with moderate hyperglycemia received 4 ml/kg row extract of ‎*C. ficifolia* fruit pulp. The average blood glucose level was 217.2 ± 30.4mg/dl at baseline. 5 h after the *C. ficifolia* ‎administration, blood glucose reached 150.8 ± 31.3 mg/dl. Hence, the results demonstrated a hypoglycemic effect by *C. ficifolia* in type 2 diabetic patients with moderate hyperglycemia.

In intensive care setting, safe and fast acting anti-diabetic agents are urgently needed to improve insulin resistance and decrease the blood glucose. The results of this preliminary study indicated that hypoglycemic effect of *C. maxima* is obvious from the first day of administration and it continued during last 3 days. However, the APACHE II and SOFA scores did not reduce in last 3 days, showing independency of the reduced blood glucose level, from improvement of illness severity in patients. In this study, none of the studied patients experienced undesirable side effects from the *C. maxima*. So, this plant seems an appropriate therapeutic to use in ICU. Further studies are needed to explore active ingredients as well as potential mechanisms of *C. maxima* responsible for its hypoglycemic effect and the mechanisms involved. Long-term larger studies are also strongly needed to confirm its hypoglycemic effects in diabetic patients admitted to ICU.

## Limitations of the study

1. The sample size calculated to be 10 pairs of subjects but we included 20 patients in our study to increase the power of study, as regards 20 patients are not enough to support clinical usage of C. maxima, we need more subjects in future studies.
2. This study was a pre-post study but of increasing the power of study we need a control group in future studies.
3. We have not checked the adverse effects of C. maxima powder in patients. Also we suggest this study to take place in patients with renal failure, liver failure and patients with active infections.
4. Stability and uniformity tests of the obtained C. maxima powder did not performed in this study.
5. Complementary analysis such as bioassay guided isolated of from anti-diabetic compounds have not been performed. Based on our results, it is suggested for to considering in future projects to evaluate pure compounds anti-diabetic effects.

## Acknowledgments

We are grateful to Shahrokh Teshnehdel and ICU staffs of Imam Reza and Shohada hospitals for their cooperation in this investigation. Applied Research Center of Tabriz University of Medical Science is greatly appreciated for supporting the study.

## Ethical Issues

The study protocol ‎was approved by the ethic committee of the Tabriz University of medical science and was recorded in the International Clinical Trials Registry Platform (ICTRP) with identifier IRCT201311118307N2.

## Conflict of Interest

The authors declared no potential conflicts of interest with respect to the research, authorship, and/or publication of this article.
